# Exploring the clinical efficacy and mechanism of high-position colon dialysis combined with Traditional Chinese Medicine retention enema in real-world patients with stage 3–5 chronic kidney disease (non-dialysis) based on the theory of the Gut–Kidney axis

**DOI:** 10.3389/fphar.2023.1246852

**Published:** 2024-01-24

**Authors:** Yanli Deng, Leixiao Zhang, Si Chen, Dongxian Xu, Wei Wu, Tao Shen, Zhen Liu, Lin Yang, Aiwei Wen, Yuhao Hou, Fanyun Shao

**Affiliations:** ^1^ Department of Nephrology, Sichuan Second Hospital of Traditional Chinese Medicine, Chengdu, China; ^2^ Division of Internal Medicine, Institute of Integrated Traditional Chinese and Western Medicine, West China Hospital, Sichuan University, Chengdu, China; ^3^ Chengdu University of Traditional Chinese Medicine, Chengdu, China

**Keywords:** gut-kidney axis, real-world study, chronic kidney diseases, high colon dialysis, Traditional Chinese medicine retention enema, indoxyl sulfate, intestinal mucosal barrier, gut microbiota

## Abstract

**Background:** With societal and economic development, the annual incidence of chronic kidney disease (CKD) is increasing. Current treatments for CKD are limited, and once patients progress to the uraemic stage, it places a significant economic burden on families and society. Based on the “gut–kidney axis” theory and real-world research, this study aims to evaluate the clinical efficacy, safety, and potential mechanism of high-position colon dialysis combined with traditional Chinese medicine (TCM) retention enema in treating stage 3–5 chronic kidney disease (non-dialysis). Additionally, it seeks to identify new therapeutic targets and approaches for CKD treatment.

**Methods:** The TCM decoction was analyzed using Ultra-Performance Liquid Chromatography-Quadrupole-Orbitrap-High Resolution Mass Spectrometry (UPLC-Q-Orbitrap-HRMS). Participants meeting the inclusion criteria were divided into a control group (n = 153) and a treatment group (n = 159) based on their preferences and physicians’ recommendations. Both groups adhered to a high-quality low-protein, low-salt, low-phosphorus, and low-fat diet supplemented with essential amino acids, and were monitored for blood pressure, blood glucose, and blood lipids. The treatment group received high-position colon dialysis combined with TCM retention enemas (administered at least 12 times every other day).

**Results:** Thirteen compounds were identified from the herbs by UPLC-Q-Orbitrap-HRMS. The CKD3–5 treatment group exhibited improvements in blood biochemistry and other laboratory indices, with significant enhancements in renal function-related indices for CKD4 and CKD5 stages (*p* < 0.05). Following treatment, indoxyl sulfate (IS), endotoxin, and D-lactic acid levels decreased to a certain extent in both groups, with a statistically significant difference observed within the treatment group (*p* < 0.05). The treatment group displayed a significant reduction in aerobic bacterial colonies, an increase in anaerobic bacterial colonies, a decrease in *Escherichia coli* colonies, and an increase in *Bifidobacterium* and *Lactobacillus* colonies (*p* < 0.05). No significant changes in colony numbers were observed in the control group.

**Conclusion:** High-position colon dialysis combined with TCM retention enema may serve as an adjuvant treatment for CKD4-5 (non-dialysis), and its mechanism may be related to the reduction of uraemic toxins, improvement of intestinal mucosal barrier function, and regulation of intestinal microecology.

**Clinical Trial Registration:** https://www.chictr.org.cn/, identifier ChiCTR2200062852.

## 1 Introduction

In recent years, there has been a global increase in the incidence of chronic kidney disease (CKD), affecting 8%–16% of the population (Stevens and Levin, 2013). Epidemiological studies have reported a prevalence of approximately 19.1% in Japanese adults (Imai et al., 2007) and 13.0% in Korean adults (Park et al., 2022). In China, the prevalence of CKD in adults is approximately 8.2% (Wang et al., 2023). As a chronic disease, CKD is often diagnosed after the onset of clinical complications or after renal function damage has occurred. Among CKD patients, 73.3%, 25.0%, and 1.8% are classified as stages 1–2, 3, and 4 to 5, respectively, with only 10.0% of people being aware of their CKD condition (Wang et al., 2023). Once kidney disease progresses, the loss of renal function becomes progressive and irreversible. Failure to intervene during the early stages of the disease leads to end-stage renal disease (ESRD), which can only be treated with renal replacement therapies such as dialysis or kidney transplantation to ensure survival, thereby imposing a significant economic burden on patients and society (Ruiz-Ortega et al., 2020).

Due to the limited available treatments for CKD, there is an urgent need for new therapeutic approaches to delay disease progression. In recent years, the “gut–kidney axis” theory has gained prominence, elucidating the intricate relationship between the kidneys and the intestines in the physiology and pathology of CKD. This theory offers novel targets and insights for future clinical interventions (Monteiro and Berthelot, 2021). High-position colon dialysis combined with retention enema and TCM has been widely used clinically as an important auxiliary treatment in China for a long time (Yan et al., 2021). Colonic dialysis, in conjunction with retention enema of TCM, is extensively utilized in clinical practice in China due to its well-established efficacy. As our understanding of the “gut-kidney” axis theory continues to advance, research has indicated that colonic dialysis can safeguard renal function in patients with pre-dialysis chronic kidney disease by modulating the gut microbiota (Li et al., 2021). Most of the existing research primarily focuses on clinical reports of colonic dialysis or TCM retention enema treatment, mainly examining biochemical indicators, without delving into the underlying mechanisms. In contrast, this real-world study is conducted within the framework of the “gut-kidney axis” theory, aiming to comprehensively evaluate the clinical efficacy and safety of high-position colon dialysis combined with TCM retention enema treatment. Additionally, the study investigates the impact of this treatment on intestinal uraemic toxins and intestinal microecology. Moreover, it delves into the potential mechanisms that contribute to the protective effects of this treatment approach on renal function, thereby addressing the existing knowledge gap in clinical practice.

## 2 Methods

### 2.1 Diagnostic criteria

• Diagnostic reference for CKD: renal impairment for ≥3 months, with or without decreased glomerular filtration rate (GFR). Renal impairment refers to structural or functional abnormalities of the kidney, manifested as one of the following: abnormal renal pathomorphology or indicators of renal damage, including abnormal blood and urine composition, or abnormal renal imaging.
• CKD3–5 staging reference: The diagnostic and staging criteria for CKD were adopted from the Kidney Disease Improving Global Outcomes (KDIGO) guidelines proposed by the International Kidney Disease Organization in 2012.

### 2.2 Inclusion, exclusion, and discontinuation criteria

#### 2.2.1 Inclusion criteria

• Age between 18 and 70 years old, without gender limitation.
• Participants who met the diagnostic criteria for CKD and the staging criteria of the KDIGO guidelines were screened for CKD3–5 (non-dialysis).
• The participants understood and agreed to participate in this study and signed an informed consent form.

#### 2.2.2 Exclusion criteria

• Presence of intestinal and anal bleeding, haemorrhoid bleeding, rectal stenosis, colitis, intestinal tumours, or other intestinal lesions.
• Patients with acute kidney injury.
• Severe allergic constitution.
• Presence of severe cardiopulmonary dysfunction, liver failure, blood system diseases, or other serious medical conditions.
• Women who are pregnant or lactating.
• Patients with severe depression, schizophrenia, or other serious psychiatric disorders.
• Inability of the participants to understand the questions posed by investigators and provide accurate responses.

#### 2.2.3 Discontinuation and drop-out criteria

• Misplacement and misacceptance.
• Poor compliance, not adhering to the prescribed treatment.
• Self-withdrawal of participants from the study.
• Participants who experienced serious adverse events during the trial or became ineligible to continue due to disease progression.
• Development of certain comorbidities, complications, or special physiological changes that rendered continued participation in the study unsuitable.

### 2.3 General information

All participants in this study were recruited from the outpatient and inpatient wards of the Department of Nephrology at the Sichuan Second Hospital of TCM between 12 May 2022 and 30 May 2023 and met the inclusion criteria for CKD stage 3–5 (non-dialysis stage). Laboratory test results and examination data were obtained from the clinical-research-chronic disease management database of the Nephrology Department of Sichuan Second Hospital of TCM and the China United Hospital Information System. This study was approved by the Clinical Ethics Committee of the Sichuan Second Hospital of TCM (Ethical Approval No: 202202-14-25).

### 2.4 Grouping method

Based on real-world research, participants who met the inclusion criteria were divided into treatment and control groups based on their preferences and doctors’ recommendations.

### 2.5 Treatment methods

#### 2.5.1 Standard treatment

Both groups followed a high-quality low-protein, low-salt, low-phosphorus, low-fat diet and received essential amino acid supplementation. Standard treatments included blood pressure and blood glucose control, blood lipid management, anaemia correction, adjustment of calcium and phosphorus metabolism disorders, syndrome differentiation, and oral Chinese medicine. Angiotensin-converting enzyme inhibitors/angiotensin receptor blockers (ACEIs/ARBs) were recommended as the first choice for antihypertensive drug control in individuals with CKD. If ACEI or ARB was not applicable for a patient due to their condition, a calcium channel blocker (CCB) was recommended. The target blood pressure for individuals with CKD was ≤130/80 mmHg (1 mmHg = 0.133 kPa).

#### 2.5.2 Intervention methods

##### 2.5.2.1 Preparation of TCM decoction for UPLC-Q-Orbitrap-HRMS

The formula of TCM decoction for colon dialysis consists of the following ingredients: *Rheum officinale* Baill (15 g, #230501), *Astragalus membranaceus* Bunge (45 g, #Y230101-2), *Salvia miltiorrhiza* Bunge (45 g, # 230524), *Oyster shell* (45 g, #230415), *Ligusticum chuanxiong* Hort (25 g, #230326), and Carthamus tinctorius L. (15 g, #230401). All Chinese medicinal herbs were provided by Sichuan Traditional Chinese Medicine Herbal Pieces Co., Ltd. The appropriate amount of sample powder was taken and extracted, filtered to obtain the sample solution. The sample solution was analyzed by UPLC-Q-Orbitrap-HRMS (Thermo Fisher Scientific, United States). The conditions as follows:

• Liquid Chromatography Conditions

Column: Thermo Scientific AccucoreTM C18 column (100 mm × 3 mm, 2.6 μm); Injection volume: 3 μL; Mobile phase: 0.1% formic acid water (A) - acetonitrile (B); Flow rate: 0.25 mL/min; Column temperature: 30°C; Gradient elution: (0–2 min, 2% B; 2–8 min, 2% →75% B; 8–11.0 min, 75% →75% B; 11.0–11.5 min, 75% →2% B; 11.5–15.0 min, 2% B).

• Mass Spectrometry Conditions

The electrospray ionization (ESI) source was used for simultaneous detection in positive and negative ion modes. The spray voltage was set to 5500 V (+) and 4500 V (-). The nebulizing gas pressure was maintained at 0.40 MPa, and the ion source temperature was set to 550°C.

##### 2.5.2.2 Preparation of TCM decoction for high-position colon dialysis

The specified Chinese medicinal herbs were soaked in 500 mL of cold water for 30 min. They were then boiled vigorously until reaching a boiling point and simmered over low heat for 20 min. The resulting first decoction, approximately 200 mL, was carefully poured into a container. This process was repeated three times. The decoctions from each round were thoroughly mixed together and stored at room temperature. The TCM decoction was uniformly prepared by the pharmacy of Sichuan Second Hospital of TCM.

##### 2.5.2.3 High-position colon dialysis combined with TCM retention enema procedure

The specific procedure for high colon dialysis combined with TCM retention enema treatment was as follows: The high colon dialysis machine (Type: IMS-100A; Manufacturer: Sanhe Bri-Prospect Medical Equipment Co., Ltd, Beijing, China) was turned on and preheated for 1–2 min of 37°C–38°C and a cavity pressure of 70 kPa. The flow rate of the main pump was 600 mL/min. The patient assumed a lateral position with raised buttocks (approximately 10°). The front end of the anal canal was lubricated with paraffin oil for approximately 10–15 cm. The thick and thin tubes were then connected and reinforced, and the end of the anal canal was connected to a sewer. The patient underwent a digital examination of the anus, which was also lubricated with paraffin oil. The head of the disposable anal tube was gently inserted 10–12 cm into the anus. The main pump was turned on, and a thin tube was inserted 40–70 cm into the patient’s body. Colonic dialysate, prepared by combining dialysate A and B with purified water in a 1:1:1.3:32 proportion, was gradually introduced deeper. The depth was increased until reaching the middle of the descending colon and the lavage fluid became clear without faecal residue. Then, high colon dialysis (planned dialysis total 15000 mL) was performed. Dialysis was stopped after approximately 30 min of adequate dialysis. After dialysis, the TCM decoction was heated to 37°C–38°C and injected into the upper colon of the patient using a drug pump. The tube was removed, and the patient was instructed to retain the solution for 1–2 h.

Participants in both groups received standard treatment. Participants were assigned to the control group, receiving standard treatment, or the treatment group, receiving high colon dialysis combined with TCM retention enema, based on their preferences and doctors’ recommendations. The treatment group received high colon dialysis combined with TCM retention at a frequency of more than 12 times every other day. The patients were followed up for 3 months.

### 2.6 Laboratory Indicators

#### 2.6.1 Laboratory-related test indicators

Fasting venous blood samples were collected from both groups before and after treatment. All blood sample indices were measured using an automatic biochemical analyser in the hospital’s laboratory department. The indicators included:

• Routine blood test: haemoglobin (Hb);
• Liver function: alanine aminotransferase (ALT), aspartate aminotransferase (AST), alkaline phosphatase (ALP), total protein (TP), albumin (ALB);
• Kidney function: urea (Urea), creatinine (CREA), uric acid (UA), cystatin C (CysC), estimated glomerular filtration rate (eGFR);
• Blood lipid: [glycerol] Triglyceride (TG), total cholesterol (TC);
• Blood electrolytes: potassium (K), calcium (Ca), inorganic phosphorus (P);

#### 2.6.2 Gut-derived uraemic toxins

Indoxyl sulfate (IS) was detected using modified enzymatic spectrophotometry. The kit was purchased from Shanghai Fengshou Biotechnology Co., Ltd., and the procedures were followed strictly as per the manufacturer’s instructions.

#### 2.6.3 Intestinal mucosal barrier function

Fasting venous blood samples (5 mL) were obtained from the participants before and after treatment and collected in heat-free test tubes. The serum was separated within 2 h. Endotoxin levels were determined using the Limulus test tripeptide matrix chromogenic method. Plasma D-lactic acid concentrations were determined using modified enzymatic spectrophotometry. The plasma D-lactic acid detection kit was purchased from Beijing Boou Shide Biotechnology Co., Ltd. The detection steps were performed according to the manufacturer’s instructions.

#### 2.6.4 Culture and detection of gut microbiota

A total of 30 g of fresh faeces were divided into three sterile test tubes, immediately sealed with a rubber stopper, and sent for testing within 15 min. After 10-fold serial dilution, the samples were inoculated onto plates at the appropriate dilution, and aerobic and anaerobic cultures were performed. Colony morphology on the surface of the Petri dishes was observed and counted. The smear was further confirmed under a light microscope, and colonies with good growth and suitable characteristics were selected. According to the dilution concentration and average number of colonies, the number of colony-forming units per gram of stool specimen was calculated, and the colony-forming unit data were converted into logarithmic values. Bacterial species were identified using internationally recognised references such as Bergey’s Bacterial Identification Manual, along with morphological staining and biochemical characteristics of the bacterial colonies. The main colonies observed in this study were aerobic (*Escherichia coli* and *Enterococcus*) and anaerobic (*Bifidobacterium* and *Lactobacillus*) bacteria.

### 2.7 Statistical analysis

The measurement data are expressed as mean ± standard deviation (X ± s). Intragroup comparisons were conducted using the paired sample t-test, while intergroup comparisons were performed using the independent sample t-test. Count data were analysed using the Chi-square test. Statistical significance was set at *P*-level <0.05.

## 3 Results

### 3.1 The chemical components of TCM decoction

**FIGURE 1:**
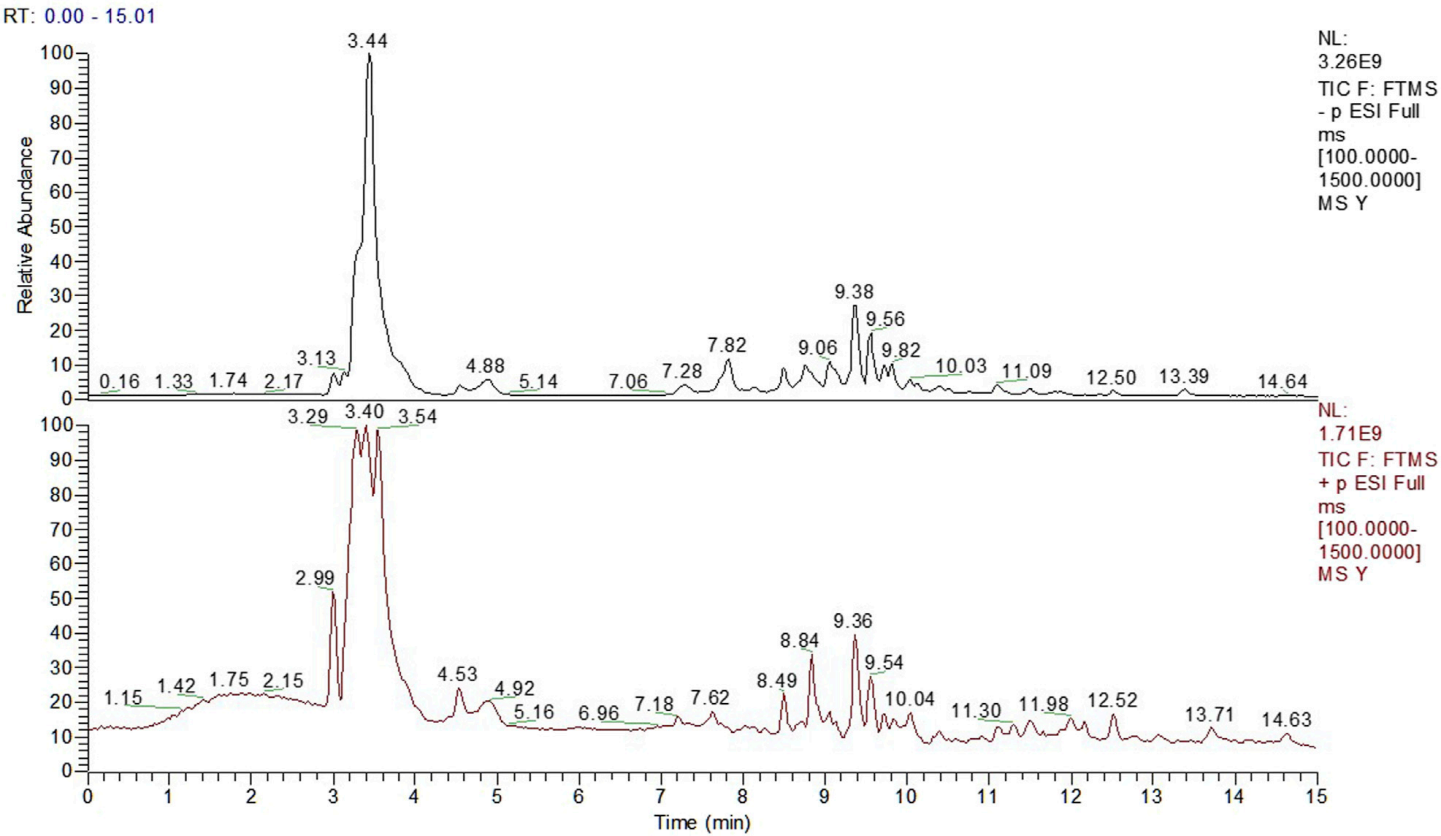
The Total Ion Chromatogram of TCM Decoction panel 1 showed the total ion chromatogram (TIC) of TCM decoction.

Figure 1 showed the total ion chromatogram (TIC) of TCM decoction. Thirteen compounds were identified from the herbs by UPLC-Q-Orbitrap-HRMS (Table 1).

**TABLE 1:** Chemical profiles of the formula.

NO.	Compound	Observed RT (min)	Adducts	M/Z	Formula
1	Tanshinone IIA	14.53	[M + H]^+^	295.13	C19 H18 O3
2	Aloe-emodin	9.72	[M + H]^+^	271.06	C15 H10 O5
3	Rheic acid	11.82	[M-H]^-^	283.03	C15 H8 O6
4	Biochanin A	11.97	[M + H]^+^	285.08	C16 H12 O5
5	Astragaloside IV	9.91	[M + H]^+^	785.47	C41 H68 O14
6	Kaempferol	9.27	[M + H]^+^	287.05	C15 H10 O6
7	Safflomin A	8.13	[M-H]^-^	611.16	C27 H32 O16
8	Ferulic acid	9.37	[M + H]^+^	195.07	C10 H10 O4
9	Hydrocinnamic acid	9.23	[M-H]^-^	149.06	C9 H10 O2
10	Cinnamic acid	9.37	[M + H]^+^	149.06	C9 H8 O2
11	Salvianolic acid A	9.55	[M-H]^-^	493.11	C26 H22 O10
12	Danshensu	7.81	[M-H]^-^	197.05	C9 H10 O5
13	Salicylic acid	8.56	[M-H]^-^	137.02	C7 H6 O3

Thirteen compounds were identified from the herbs by UPLC-Q-Orbitrap-HRMS.

### 3.2 Baseline characteristics of patients with CKD3–5 stages

A total of 317 patients were enrolled in this study, with 153 in the control group and 164 in the treatment group. During the course of the study, five patients from the treatment group withdrew due to intolerance factors such as perianal disease and disease progression. Ultimately, 312 patients successfully completed the study. Baseline data, including sex, age, blood pressure, primary kidney disease, routine blood tests, liver and kidney function, electrolytes, and other potential confounding factors that could affect disease progression or clinical efficacy, were recorded. The patients were further stratified based on their eGFR. There were no significant differences between the control group (n = 153) and the treatment group (n = 159) in baseline data (*p* > 0.05) (Tables 2, 3, and 4).

**TABLE 2:** Baseline characteristics of patients of stage 3 CKD.

Group	Sex male/female	Age/year	Course of disease/year	Urea mmol/L	Scr umol/L	UA umol/L	CysC mg/L	eGFR mL/min	K mmol/L	Ca mmol/L
Control Group (n = 50)	26/24	52 ± 18.36	6.41 ± 4.89	10.12 ± 2.79	156.30 ± 19.34	419.30 ± 108.89	2.22 ± 0.88	45.41 ± 8.47	4.34 ± 0.73	2.35 ± 0.23
Treatment Group (n = 48)	21/27	55 ± 15.58	6.17 ± 5.23	9.92 ± 2.88	157.17 ± 24.10	436.42 ± 91.72	1.91 ± 0.42	43.53 ± 12.09	4.54 ± 0.43	2.37 ± 0.18
P	0.23	0.95	0.81	0.79	0.89	0.53	0.09	0.65	0.20	0.73

Group	ALT U/L	AST U/L	ALP U/L	TP g/L	ALB g/L	HGB g/L	TG mmol/L	TC mmol/L	P mmol/L	PTH pg/mL
Control Group (n = 50)	21.74 ± 10.32	22.00 ± 7.57	78.22 ± 32.95	69.52 ± 7.95	41.38 ± 3.92	128.00 ± 17.36	2.20 ± 1.14	4.29 ± 1.69	1.21 ± 0.18	106.57 ± 36.34
Treatment Group (n = 48)	28.31 ± 17.61	26.53 ± 10.07	83.97 ± 21.76	69.27 ± 6.58	41.57 ± 4.15	131.89 ± 16.66	2.35 ± 1.23	4.94 ± 1.10	1.28 ± 0.19	129.79 ± 54.82
P	0.12	0.07	0.43	0.90	0.87	0.40	0.65	0.09	0.18	0.08

**TABLE 3:** Baseline characteristics of patients of stage 4 CKD.

Group	Sex male/female	Age/year	Course of disease/year	Urea mmol/L	Scr umol/L	UA umol/L	CysC mg/L	eGFR mL/min	K mmol/L	Ca mmol/L
Control Group (n = 52)	27/25	64 ± 17.43	7.41 ± 5.89	17.85 ± 7.54	269.83 ± 32.06	411.16 ± 90.08	3.37 ± 1.22	21.68 ± 3.63	4.57 ± 0.72	2.34 ± 0.25
Treatment Group (n = 55)	30/25	62 ± 19.76	8.17 ± 3.23	16.84 ± 6.71	277.43 ± 43.21	408.55 ± 123.99	3.24 ± 0.86	21.54 ± 4.04	4.67 ± 0.60	2.43 ± 0.40
P	0.56	0.61	0.68	0.67	0.57	0.95	0.72	0.81	0.65	0.47

Group	ALT U/L	AST U/L	ALP U/L	TP g/L	ALB g/L	HGB g/L	TG mmol/L	TC mmol/L	P mmol/L	PTH pg/mL
Control Group (n = 52)	19.33 ± 11.02	24.07 ± 10.29	75.93 ± 25.08	62.59 ± 6.63	37.40 ± 5.68	96.47 ± 24.14	2.59 ± 2.04	4.55 ± 1.32	1.48 ± 0.14	238.07 ± 65.24
Treatment Group (n = 55)	17.13 ± 7.31	21.54 ± 4.04	90.63 ± 32.63	66.64 ± 7.24	40.28 ± 3.99	97.83 ± 9.97	2.28 ± 1.06	4.68 ± 1.42	1.47 ± 0.18	217.58 ± 140.98
P	0.47	0.30	0.16	0.13	0.08	0.81	0.54	0.78	0.90	0.61

**TABLE 4:** Baseline characteristics of patients of stage 5 CKD.

Group	Sex male/female	Age/year	Course of disease/year	Urea mmol/L	Scr umol/L	UA umol/L	CysC mg/L	eGFR mL/min	K mmol/L	Ca mmol/L
Control Group (n = 51)	26/25	69 ± 11.91	7.97 ± 6.89	26.02 ± 4.40	489.55 ± 122.89	427.68 ± 121.09	4.28 ± 0.71	9.80 ± 2.42	4.31 ± 0.70	2.23 ± 0.24
Treatment Group (n = 56)	30/26	68 ± 15.37	8.47 ± 7.21	25.74 ± 12.60	499.98 ± 102.28	453.01 ± 143.11	3.71 ± 1.07	10.28 ± 2.37	4.69 ± 0.57	2.25 ± 0.21
P	0.34	0.83	0.54	0.94	0.77	0.57	0.08	0.66	0.06	0.82

Group	ALT U/L	AST U/L	ALP U/L	TP g/L	ALB g/L	HGB g/L	TG mmol/L	TC mmol/L	P mmol/L	PTH pg/mL
Control Group (n = 51)	15.77 ± 7.57	20.69 ± 8.77	68.54 ± 22.44	61.97 ± 8.12	37.17 ± 4.80	96.77 ± 10.63	2.68 ± 1.67	4.89 ± 0.90	1.67 ± 0.27	229.71 ± 83.37
Treatment Group (n = 56)	13.23 ± 8.73	16.92 ± 6.49	80.38 ± 35.14	64.18 ± 8.06	39.03 ± 4.59	96.00 ± 19.77	2.29 ± 1.37	4.62 ± 1.10	1.61 ± 0.30	224.83 ± 133.22
P	0.36	0.11	0.26	0.40	0.22	0.89	0.42	0.43	0.54	0.90

### 3.3 Comparison of Laboratory Indicators in CKD3–5 stages

#### 3.3.1 Comparison of Laboratory Indicators in Stage 3 CKD

**FIGURE 2:**
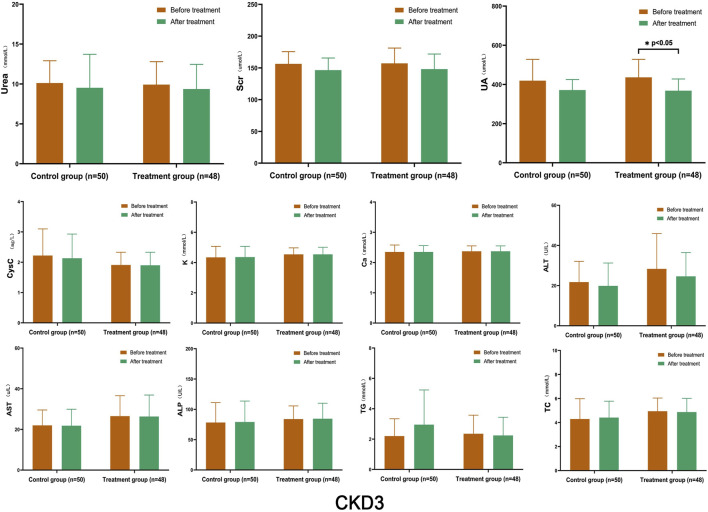
Comparison of Laboratory Indicators in Stage 3 CKD After treatment, both the control and treatment groups showed improvements in the levels of Urea, Scr, and CysC compared to before treatment; however, there was no significant difference between the two groups before and after treatment. The only significant difference was observed in the treatment group, where the UA decreased significantly after treatment (*p* < 0.05). (Before and after comparisons within the group*: *p* < 0.05).

After treatment, there was a certain degree of improvement in the levels of Urea, Scr, and CysC in both the control and treatment groups compared to before treatment. The levels of K, Ca, ALT, AST, ALP, TG, and TC remained stable, and there was no significant difference between the two groups before and after treatment. However, the UA index in the treatment group showed a significant decrease after treatment, which was statistically significant (*p* < 0.05) (Figure 2).

#### 3.3.2 Comparison of Laboratory Indicators in Stage 4 CKD

**FIGURE 3:**
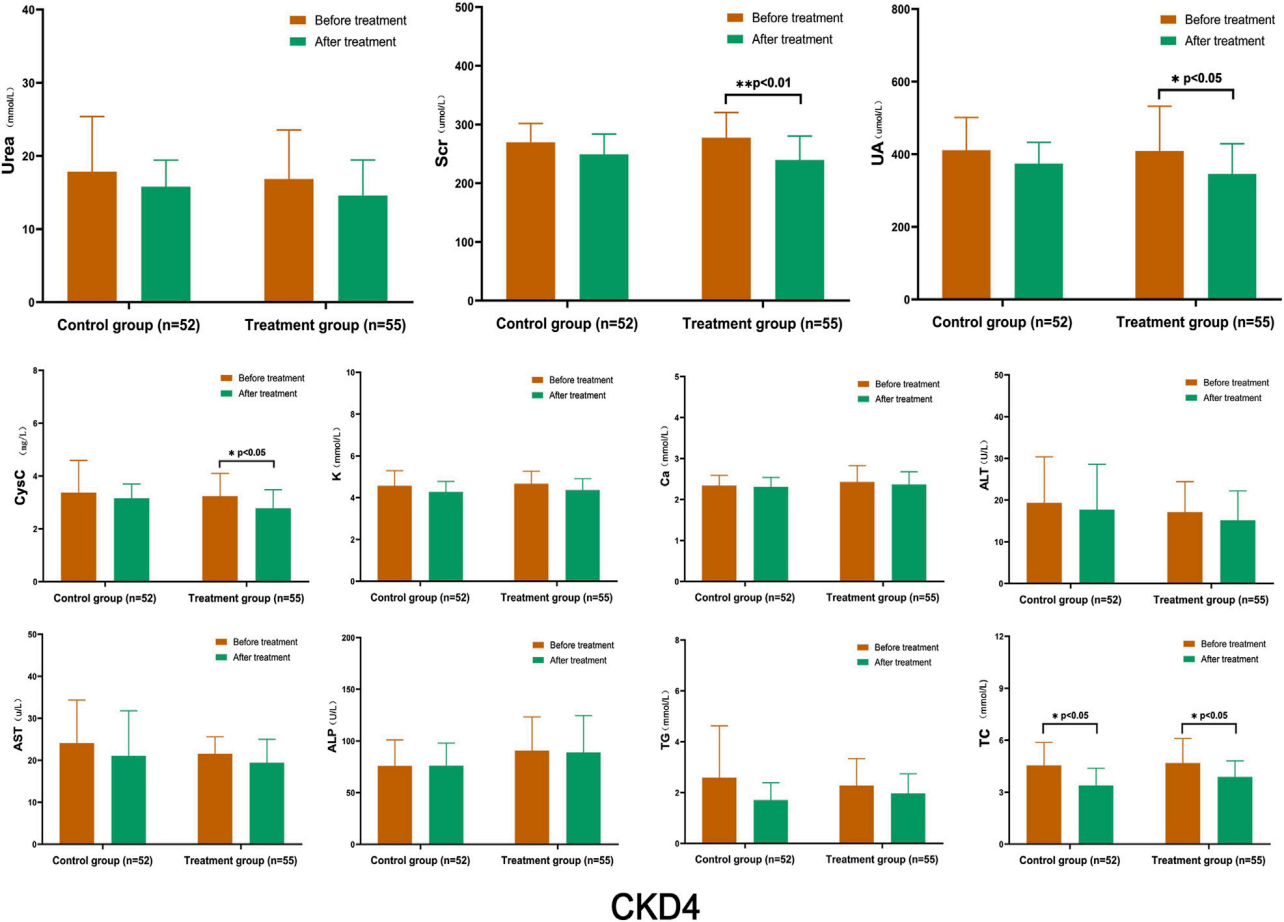
Comparison of Laboratory Indicators in Stage 4 CKD After treatment, the levels of Urea, Scr, UA, and CysC in the control group decreased to a certain extent, but the changes were not statistically significant. However, the levels of TC in the control group decreased significantly (*p* < 0.05). In the treatment group, UA, CysC, and TC levels decreased significantly (*p* < 0.05), and Scr decreased significantly (*p* < 0.01). (Before and after comparisons within the group*: *p* < 0.05; **:*p* < 0.01).

After treatment, the levels of Urea, Scr, UA, and CysC in the control group decreased to a certain extent, but the changes were not statistically significant. Conversely, the TC levels in the control group decreased significantly (*p* < 0.05). In the treatment group, UA, CysC, and TC levels decreased significantly (*p* < 0.05), and Scr decreased significantly (*p* < 0.01). The K, Ca, ALT, AST, ALP, and TG levels remained stable, and no significant changes were observed between the groups (Figure 3).

#### 3.3.3 Comparison of Laboratory Indicators in Stage 5 CKD

**FIGURE 4:**
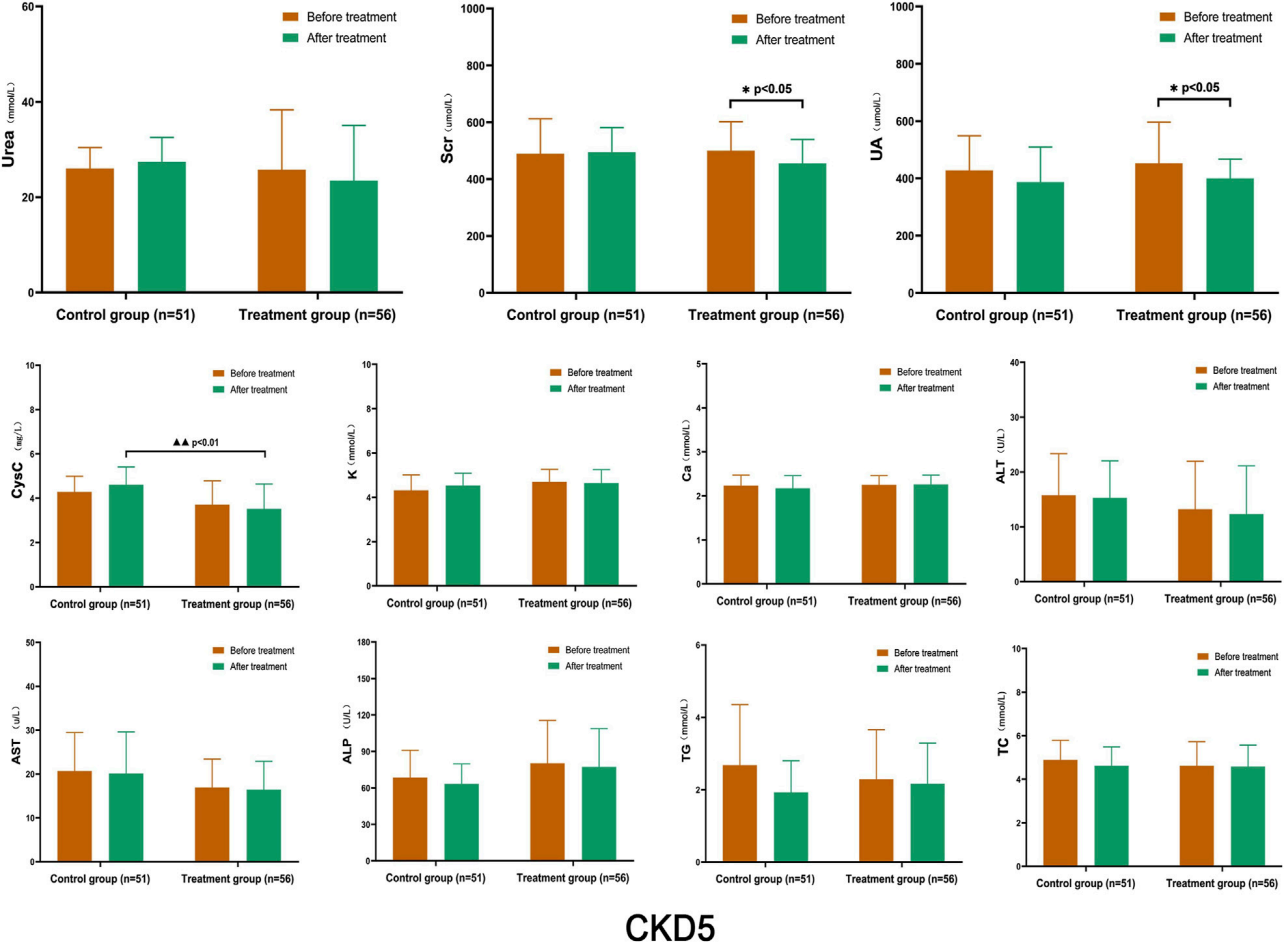
Comparison of Laboratory Indicators in Stage 5 CKD After treatment, the levels of Urea, Scr, and CysC in the control group were slightly higher than those before treatment, while UA levels were lower; however, there was no statistically significant difference within the group. In the treatment group, the levels of Urea, Scr, and UA decreased to some extent, and those of Scr and UA decreased significantly within the group (*p* < 0.05). CysC levels, when compared with the control group after treatment, showed a statistically significant difference between the two groups (*p* < 0.01). (Before and after comparisons within the group*: *p* < 0.05; Comparison between groups▲▲: *p* < 0.01).

After treatment, the levels of Urea, Scr, and CysC in the control group showed a slight increase compared to before treatment, while UA levels were lower; however, there was no statistically significant difference within the group. In the treatment group, the levels of Urea, Scr, and UA decreased to some extent, and those of Scr and UA decreased significantly within the group (*p* < 0.05). When comparing CysC levels with the control group after treatment, there was a statistically significant difference between the two groups (*p* < 0.01). The levels of K, Ca, ALT, AST, ALP, TG, and TC remained stable, and there were no significant differences between the groups (Figure 4).

#### 3.3.4 Change trend of TP, ALB, HGB, P, and PTH in CKD3–5 stages

**FIGURE 5:**
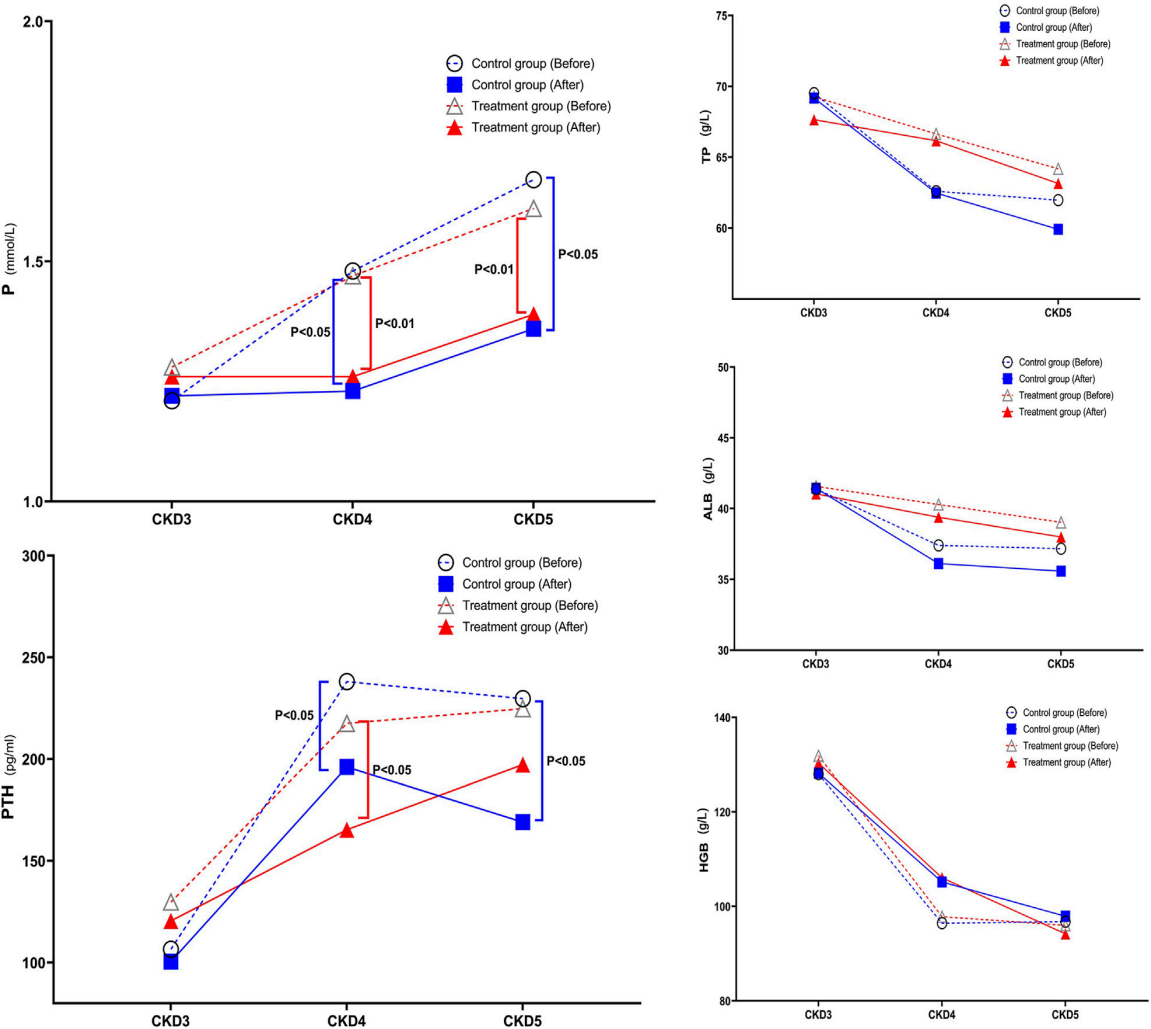
Change in Trend of TP, ALB, HGB, P, and PTH in Stage 3–5 CKD After treatment, there were no significant changes in TP, ALB, and HGB levels compared to before treatment. However, P and PTH levels showed significant improvements after treatment. In the stratified comparison, both the control and treatment groups in CKD4 and CKD5 showed significantly lower P levels after treatment compared to before treatment. The control group had a statistically significant difference before and after treatment (*p* < 0.05), and the treatment group had a statistically significant difference before and after treatment (*p* < 0.01). There was no statistically significant difference between the two groups after treatment. In the stratified comparison, PTH levels in the control and treatment groups in the CKD4 and CKD5 stages decreased to a certain extent after treatment, and the difference between the two groups in the CKD4 stage after treatment was statistically significant (*p* < 0.05). In the control group, there was a significant difference in the CKD5 stage before and after treatment (*p* < 0.05).

With further disease progression, TP, ALB, and HGB levels gradually decreased, while P and PTH levels gradually increased, which is consistent with the model of disease development. After treatment, there were no significant changes in TP, ALB, and HGB levels compared to before treatment. However, there were significant improvements in P and PTH levels compared to before treatment. In the stratified comparison, the P levels in CKD4 and CKD5 of both the control and the treatment group were significantly lower after treatment than those before treatment. The control group had statistically significant differences before and after treatment (*p* < 0.05), and the treatment group had a statistically significant difference before and after treatment (*p* < 0.01). There were no statistically significant differences between the two groups after treatment. In the CKD4 and CKD5 stages, the PTH levels, decreased to a certain extent after treatment in both the control group and the treatment group, and the difference between the two groups in the CKD4 stage after treatment was statistically significant (*p* < 0.05). In the control group, there was a significant difference in the CKD5 stage before and after treatment (*p* < 0.05) (Figure 5).

### 3.4 Comparison of serum enterogenous uraemic toxin, intestinal mucosal barrier function, and intestinal microecology in CKD3–5

In the control and treatment groups, participants in the CKD3–5 stages were randomly selected, representing 25% of the cases, to study IS, endotoxin, and D-lactic acid levels and intestinal microecology. The aim was to further explore the changing trend of the above indices in CKD3–5 stage participants and the potential mechanism of high colon dialysis combined with TCM retention enema. Due to the small number of participants (38 in the control group and 39 in the treatment group), stratified comparisons were not performed.

#### 3.4.1 Comparison of IS, endotoxin, and D-lactic acid in CKD3–5 stages

**FIGURE 6:**
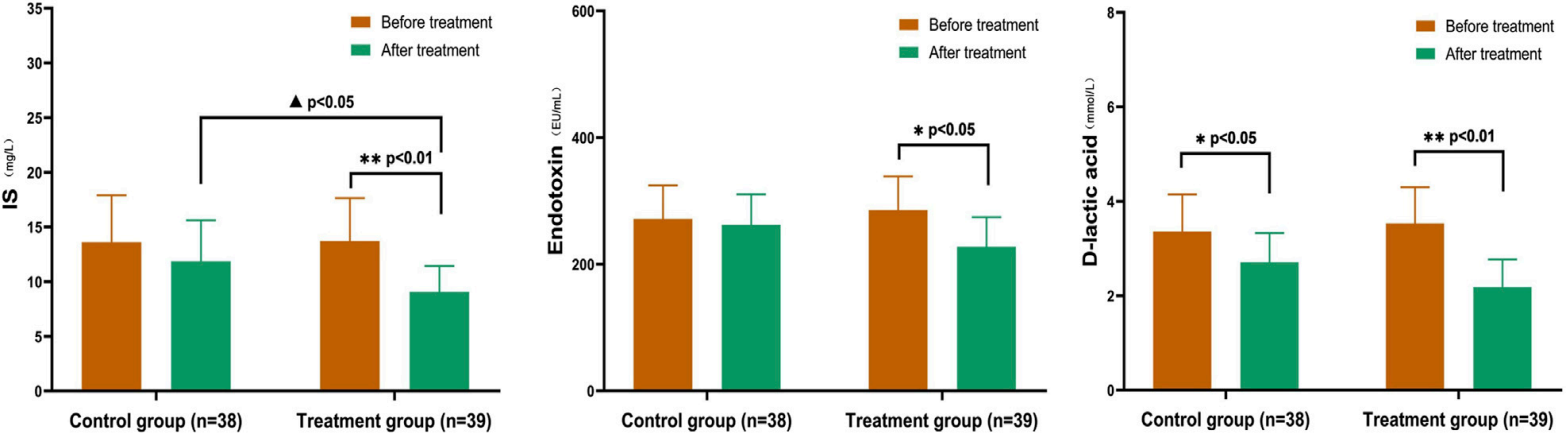
Comparison of IS, Endotoxin, and D-lactic acid in CKD3–5 Stages In the treatment group, there were significant differences in IS and D-lactic acid levels (*p* < 0.05), as well as endotoxin levels (*p* < 0.01). The difference between the two groups after IS treatment was statistically significant (*p* < 0.05). After treatment, the D-lactic acid levels before and after group comparison were statistically significant (*p* < 0.05). (Before and after comparisons within the group*: *p* < 0.05; **: *p* < 0.01; Comparison between groups▲:*p* < 0.05).

After treatment, both groups showed a decrease in IS, endotoxin, and D-lactic acid levels to a certain extent. There were significant differences in IS and D-lactic acid (*p* < 0.05), as well as endotoxin levels (*p* < 0.01), in the treatment group. The difference between the two groups after IS treatment was statistically significant (*p* < 0.05). Additionally, the D-lactic acid levels before and after treatment within the treatment group differed significantly (*p* < 0.05) (Figure 6).

#### 3.4.2 Comparison of Gut Microbiota in CKD3–5 stages

**FIGURE 7:**
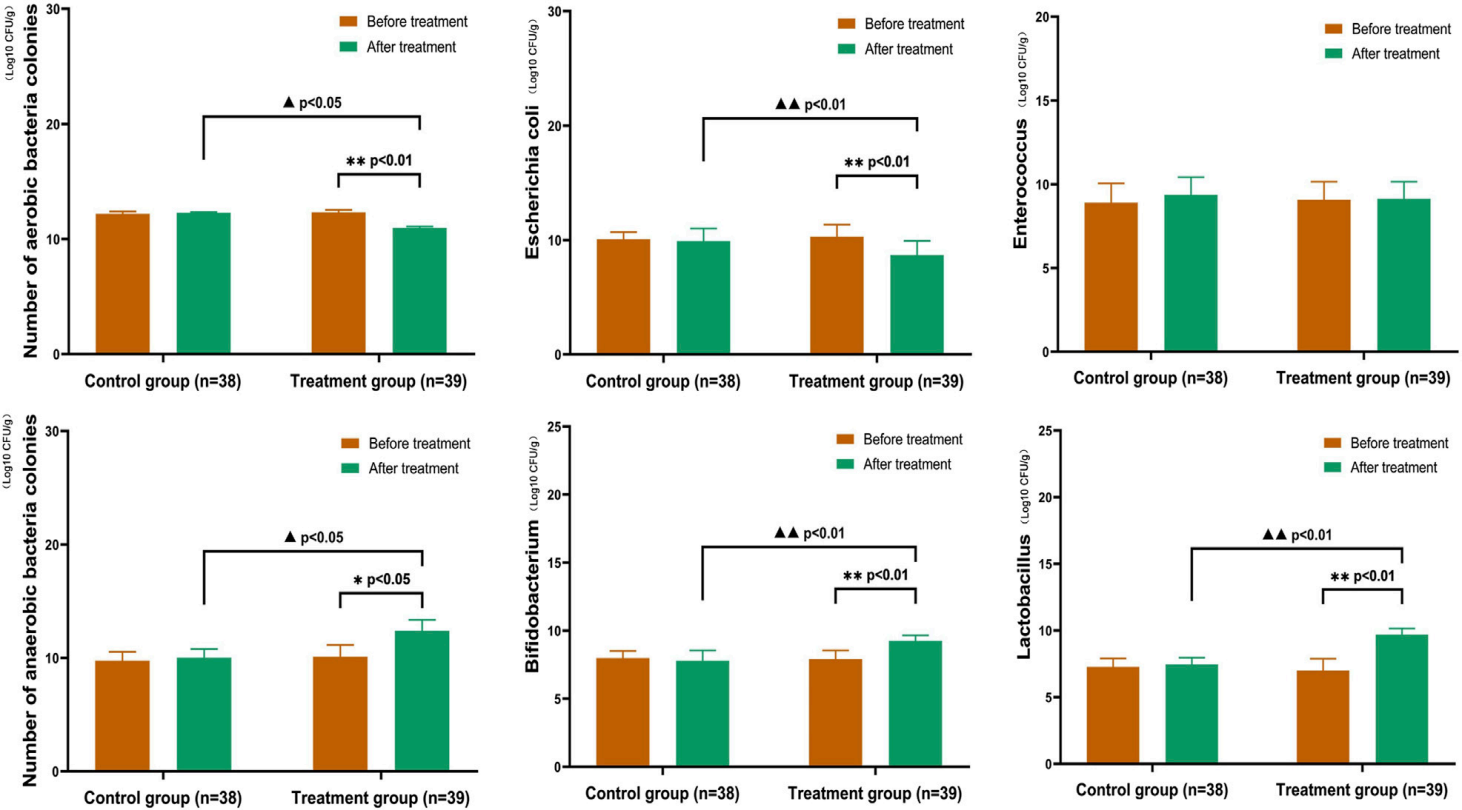
Comparison of Gut Microbiota in CKD3–5 Stages In the treatment group, the number of aerobic bacterial colonies (*Escherichia coli*) and anaerobic bacterial colonies (*Bifidobacterium* and *Lactobacillus*) showed significant differences after treatment (*p* < 0.05). After treatment, the number of aerobic bacterial colonies (*Escherichia coli*) and anaerobic bacterial colonies (*Bifidobacterium* and *Lactobacillus*) were significantly different between the two groups (*p* < 0.05). (Before and after comparisons within the group*: *p* < 0.05; **:*p* < 0.01; Comparison between groups▲:*p* < 0.05; ▲▲:*p* < 0.01).

After treatment, the treatment group showed a significant reduction in the number of aerobic bacterial colonies, an increase in the number of anaerobic bacterial colonies, a significant decrease in the number of *E. coli* colonies, and a significant increase in the number of *Bifidobacterium* and *Lactobacillus* colonies. No significant change in the number of colonies was observed in the control group. In the treatment group, the numbers of aerobic bacterial colonies (*E. coli*) and anaerobic bacterial colonies (*Bifidobacterium* and *Lactobacillus*) after treatment were significantly different (*p* < 0.05). After treatment, the numbers of aerobic bacterial colonies (*E. coli*) and anaerobic bacterial colonies (*Bifidobacterium* and *Lactobacillus*) were significantly different between the two groups (*p* < 0.05) (Figure 7).

## 4 Discussion

### 4.1 Gut–kidney axis theory

Currently, several theories exist regarding chronic renal failure, including the “glomerular hemodynamic change theory”, “podocyte injury theory”, and “transforming growth factor-β overexpression theory”. In 2011, Ritz proposed the “gut–kidney axis” theory (Meijers and Evenepoel, 2011), which encompasses two main aspects. Firstly, the kidney function of CKD patients deteriorates gradually during the progression of the disease, leading to accumulation of many metabolites in the body due to inadequate excretion. These aggregated substances are known as uraemic toxins (UTs). Uraemic toxins can disrupt the tight junction barrier of the intestinal epithelium and penetrate the intestinal lumen through the blood vessels of the intestinal wall. Consequently, this process results in disorders of the intestinal microecosystem, altering the composition and metabolism of intestinal microorganisms. As a consequence, beneficial bacteria decrease, while pathogenic bacteria increase in the intestinal tract. However, the decrease in intestinal flora diversity and the increase in intestinal pathogenic bacteria eventually contribute to an imbalance of intestinal microecosystems. This imbalance leads to the generation of gut-derived uraemic toxins (GDUTs) through the proteolysis of undigested proteins retained in the gut, leading to impairment of the intestinal epithelial barrier function. With an increase in intestinal epithelial cell permeability, GDUTs and opportunistic pathogens are more likely to enter the blood circulation, which in turn activates the intestinal mucosal immune system and triggers a systemic inflammatory response, leading to a renal microinflammatory state and aggravating the progression of CKD. Uraemic toxins are metabolised by intestinal microorganisms and interact with each other to form a vicious circle. In 2015, the concept of the “chronic kidney disease-colon axis” was reintroduced (Pahl and Vaziri, 2015), highlighting the pivotal role of gut microbiota in the accumulation of uraemic toxins, particularly through the production of large amounts of uraemic solutes during protein fermentation. This theory reveals the subtle physiological and pathological relationship between intestinal microecology and CKD. Regulating the intestinal microflora in patients with CKD may be an effective way to reduce uraemic toxin accumulation and improve the renal microinflammatory state. This study revealed elevated levels of IS, endotoxin, and D-lactic acid in patients with CKD stages 3–5 prior to treatment. These findings suggest that as eGFR decreases, there is a notable increase in GDUTs within the patients’ bodies, which supports the aforementioned theory. After undergoing treatment, a significant reduction in IS, endotoxin, and D-lactic acid was observed compared to pre-treatment levels, with statistical significance observed in the treatment group. Taking into account the alterations in aerobic and anaerobic bacteria in the treatment group, it can be further hypothesized that the substantial decrease in intestinal-derived uremic toxins among patients in the treatment group may be attributed to the modulation of the intestinal microbiota and microenvironment through the combination of high-colon dialysis and TCM enema.

### 4.2 IS, endotoxin, D-lactic acid and gut microbiota

The European Uremic Toxin Working Group has classified uremic toxins (UTs) into three categories according to their physicochemical properties: free water-soluble low-molecular-weight solutes (<500 Da), protein-binding solutes, and medium molecules (≥500 Da) (Duranton et al., 2012). UTs are primarily derived from proteins metabolised by intestinal microorganisms and converted by the liver, mitochondria, or other enzymes (Lu et al., 2021). Enterogenous uraemic toxins produced by intestinal microbiota mainly include IS, P-cresol sulfate (PCs), and trimethylamine oxide (TMAO). These gut-derived uraemic toxins are challenging to remove even in patients undergoing haemodialysis (Feroze et al., 2012). Studies have demonstrated associations between solutes such as IS and cresyl sulfate with CKD progression, cardiovascular disease, and overall mortality (Evenepoel et al., 2009; Meyer and Hostetter, 2012). In this study, IS was selected as the evaluation index for enterogenous uraemic toxins. The intestinal mucosal barrier serves as the main barrier between the body and the outside world, which not only prevents the invasion of harmful substances from the outside world but also prevents the loss of substances from the body (Odenwald and Turner, 2017). GDUTs can damage the intestinal mucosal barrier, and endotoxins and D-lactic acid serve as two important indicators of intestinal mucosal barrier function. As the patient’s condition worsens, endotoxin and D-lactic acid levels gradually increase, intestinal mucosal barrier function gradually declines, the intestinal microecological disorder occurs, and transplantation of UTs into the blood stimulates the release of cytokines, inflammatory factors, oxygen free radicals, and other harmful substances, leading to systemic inflammation and further disease aggravation (Lau et al., 2015).

The gut microbiota consists of over 100 trillion microbial cells (Ramezani and Raj, 2014). The human gut is inhabited by an extremely complex and dynamic group of bacteria that has a profound impact on human health and disease states (Ramezani et al., 2016). The intestinal microecological disorder in patients with CKD is particularly significant in the colon, characterized by excessive proliferation of aerobic bacteria such as *E. coli* and a significant reduction in anaerobic bacteria like *Bifidobacterium* and *Lactobacillus*. The extent of this flora imbalance is closely related to the severity of renal failure, systemic inflammation, and nutritional status, especially in stage 5 (Poesen et al., 2016; Rysz et al., 2021). This study demonstrated a potential positive correlation between IS, endotoxin, and D-lactic acid levels and CKD progression. These results also reaffirm this viewpoint. Clinical symptoms related to GDUT accumulation, such as nausea and loss of appetite, became more pronounced. Participants receiving high colon dialysis combined with TCM retention enema showed more significant improvements in IS levels, intestinal mucosal barrier function, and relief of clinical symptoms compared to the control group. In the comparative analysis of CKD stages, it was observed that patients in CKD stages 4–5 exhibited a gradual decline in HGB levels, TP, and ALB, while P and PTH levels showed a gradual increase compared to CKD stage 3 patients. Additionally, patients in CKD stages 4–5 experienced worsening disruptions in calcium-phosphate metabolism and symptoms of malnutrition. Particularly in CKD stage 5 patients, the imposition of dietary restrictions was significantly associated with disruptions in calcium-phosphate metabolism, which may contribute to intestinal dysbiosis. While there are currently effective medications available for regulating calcium-phosphate and PTH levels, there is a lack of effective drugs for improving malnutrition symptoms. The trial results suggest that although high-position colon dialysis combined with traditional Chinese medicine enema may not directly improve indicators such as anaemia and hypoalbuminemia, this treatment approach has the potential to regulate the intestinal microenvironment and promote the growth of beneficial probiotic colonies. This, in turn, could potentially enhance nutrient absorption and ameliorate malnutrition symptoms in CKD patients.

### 4.3 High-colon dialysis combined with TCM retention enema

High-colon dialysis combined with TCM retention enema is a widely used external treatment with TCM characteristics in the clinical management of CKD in China. A clinical study demonstrated that high colon dialysis can effectively lower serum creatinine levels and improve renal function in CKD4-5 patients, thereby serving as an adjuvant therapy to delay disease progression (Dai et al., 2019). Numerous clinical trials have shown that high colon dialysis combined with TCM retention enema can not only effectively improve clinical symptoms and reduce indicators such as serum creatinine and uric acid but also effectively address CKD and bone mineral metabolism disorders (Zhou et al., 2014; Yan et al., 2022; Zhang et al., 2022). The intestinal dialysis prescription, developed by Professor Wu Kang-heng based on his long-term clinical experience, has been employed in the treatment of CKD in our department for several decades. Previous clinical studies have shown that this regimen effectively delays disease progression and improves renal function (Shen et al., 2015). Modern pharmacology indicates that the granules of *R. officinale Baill* can regulate the intestinal microecology in CKD rats, promoting the restoration of intestinal microecology balance, which mainly involves regulating intestinal flora (increasing the probiotic *Bifidobacterium* and reducing the pathogenic bacteria *Enterobacter* and *Enterococcus*), increasing the expression of tight junction proteins and occludin in the intestinal mucosa, and reducing the levels of GDUTs such as IS and endotoxin lipopolysaccharide (Luo et al., 2020). *Astragalus membranaceus* Bunge combined with *S. miltiorrhiza* Bunge can regulate the NF-κB/HIF-α signalling pathway, downregulate the expression of P65, p-P65, and HIF-1α, inhibit the mesenchymal transition of renal tubular epithelial cells, and reduce the deposition of extracellular matrix and collagen fibres, thereby delaying the process of renal interstitial fibrosis (Qin, 2021). Research has shown that the main component of *L. chuanxiong* Hort, ferulic acid, can remodel the composition of the gut microbiota, characterized by an increase in beneficial bacteria (e.g., *Lactobacillus and Ruminococcus*) and a decrease in pathogenic bacteria (e.g., *Bacteroides*) (Zhang et al., 2023). Although current research on these drugs as primary components of enema prescriptions is mainly based on oral administration, research on rectal administration is relatively scarce. Further research is necessary to determine whether the effects of rectal administration are equivalent to those of oral administration.

### 4.4 Potential underlying mechanisms

We speculate that the potential mechanisms underlying the beneficial effects of high colon dialysis combined with TCM retention enema treatment on CKD may be as follows: First, the uraemic patients have much higher daily intestinal solutes compared to those with normal urine (Sparks, 1979). Large amounts of colon-derived uraemic toxins contribute to CKD progression. High colon dialysis aids in removing these toxins from the gut by promoting defaecation. Second, colonic dialysis operates on the principle of peritoneal dialysis, utilising transmembrane transport mechanisms, such as penetration, diffusion, and absorption. This approach takes advantage of the concentration differences between the cells and the outside to transport transmembrane substances (Wei et al., 2014). Using a colon dialysis machine, the TCM liquid reaches the high colon area. The intestinal mucosa acts as a barrier, and with the semi-permeability of the intestinal mucosa, approximately 3/4 of the liquid enters the human blood circulation directly through the anal vein and subintimal vein of the rectum, bypassing the liver, which avoids the first-pass metabolism and improves the drug absorption in the body (Pham et al., 2008; Yang, 2016). Further removal of residual toxins and repair of the intestinal mucosal barrier aim to restore the intestinal state of CKD patients as close to normal as possible (Ulluwishewa et al., 2011).

The results of this study demonstrated a certain degree of improvement in Urea, Scr, and CysC levels in both groups of CKD stage 3 patients after treatment. However, these improvements were not statistically significant, which could be attributed to the relatively mild condition of CKD stage 3 patients and their partially compensatory kidney function. On the other hand, in CKD stage 4–5 patients, the treatment group exhibited a significant and statistically meaningful reduction in Scr and CysC levels compared to pre-treatment values.

Based on these findings, it can be inferred that the combination of high-position colon dialysis and traditional Chinese medicine enema effectively eliminated the accumulation of IS, endotoxins, and D-lactic acid in the intestines. This approach also increased the population of *Bifidobacterium* and *Lactobacillus*, thereby improving the disrupted intestinal microbiota. Consequently, it reduced toxin accumulation in the body, ameliorated the microinflammatory state commonly observed in CKD patients, and lowered Urea, Scr, and CysC levels. These beneficial effects ultimately contribute to the preservation of kidney function through multiple mechanisms.

Importantly, this treatment regimen demonstrated a high level of safety without significant hepatorenal toxicity, hematotoxicity, or electrolyte disturbances. It was also well-tolerated by patients, suggesting that it could potentially serve as a novel strategy and approach for managing stage 4–5 CKD.

### 4.5 Data processing

The key to establishing evidence-based practice in real-world research on the treatment of chronic kidney disease with TCM lay in eliminating the confounding factors that might have influenced the evaluation of treatment effectiveness. The team was composed of specialist physicians as the main project team members. Roles and responsibilities were clearly defined, and standardized diagnostic and treatment procedures were followed to ensure quality control throughout the entire process, including patient enrolment and treatment. Additionally, we had dedicated personnel responsible for data entry and verification, actively monitoring and identifying any potential anomalies that might have arisen during the trial. These effective measures ensured the maximum reliability of the data source and minimized confounding factors. Depending on the type of data and research objectives, various statistical methods such as propensity score matching, multivariate analysis, and instrumental variable analysis were used to control for confounding factors. These methods helped reduce biases and corrected for known and potential confounders at various stages of the study, providing further assurance for the reliability of the data.

## 5 Conclusion

High-colon dialysis combined with TCM retention enema may serve as an adjuvant treatment for CKD4-5 (non-dialysis) patients, and its mechanism could involve the reduction of uraemic toxins, improvement of intestinal mucosal barrier function, and regulation of intestinal microecology. Despite our best efforts in patient recruitment and maintaining a rigorous approach to the trial procedures and results, the total number of patients in our study was still significantly lower compared to other large-scale real-world studies. Additionally, we lacked a multi-centre case source, which introduced limitations and biases to our findings. However, it was encouraging to note that our study had still identified some beneficial treatment trends. This motivated us to continue collecting relevant data in clinical practice, aiming to provide a more objective evaluation of the effectiveness and safety of TCM in the treatment of CKD.

## Data Availability

The original contributions presented in the study are included in the article/Supplementary material, further inquiries can be directed to the corresponding author.
